# Liposarcoma cells with aldefluor and CD133 activity have a cancer stem cell potential

**DOI:** 10.1186/2045-3329-1-8

**Published:** 2011-08-01

**Authors:** Eva W Stratford, Russell Castro, Anna Wennerstrøm, Ruth Holm, Else Munthe, Silje Lauvrak, Bodil Bjerkehagen, Ola Myklebost

**Affiliations:** 1 Cancer Stem Cell Innovation Centre and Department of Tumor Biology, Institute of Cancer Research, Oslo University Hospital, The Norwegian Radium Hospital, PO Box 4953 Nydalen, Oslo, NO-0424, Norway; 2 Department of Pathology, Oslo University Hospital, The Norwegian Radium Hospital, PO Box 4953 Nydalen, Oslo, NO-0424, Norway; 3 Department of Molecular Bioscience, University of Oslo, PO-Box 1041 Blindern, Oslo, NO-0316, Norway

## Abstract

Aldehyde dehydrogenase (ALDH) has recently been shown to be a marker of cancer stem-like cells (CSCs) across tumour types. The primary goals of this study were to investigate whether ALDH is expressed in liposarcomas, and whether CSCs can be identified in the ALDH^high ^subpopulation. We have demonstrated that ALDH is indeed expressed in 10 out of 10 liposarcoma patient samples. Using a liposarcoma xenograft model, we have identified a small population of cells with an inducible stem cell potential, expressing both ALDH and CD133 following culturing in stem cell medium. This potential CSC population, which makes up for 0, 1-1, 7% of the cells, displayed increased self-renewing abilities and increased tumourigenicity, giving tumours *in vivo *from as few as 100 injected cells.

## Introduction

CSCs are described as a small population of tumour cells possessing stem-like properties, such as the ability to self-renew, as well as to differentiate into more mature cells that make up the bulk of the tumour, which usually to some extent resembles normal tissue. These cells are also referred to as tumour initiating [1].

The CSCs are in many aspects similar to normal stem cells, and are thought to arise either when normal stem cells gain oncogenic mutations, which confer enhanced proliferation and lack of homeostatic control mechanisms, or alternatively when a progenitor or differentiated cell acquires mutations conferring de-differentiation to a malignant stem-like cell [2]. Since the integrity of stem cells is of critical importance for the organism, several mechanisms that ensure the survival of stem cells have evolved. These mechanisms include enhanced activity of membrane pumps which remove toxic substances [3], and enhanced activity of enzymes such as aldehyde dehydrogenase (ALDH), which confer resistance to toxic agents [4,5]. ALDH1 was also found to be implicated in regulating the stem cell fate in hematopoietic stem cells (HSCs) [6]. Properties and functions of normal stem cells can also be employed to enrich CSCs. In this respect, the Aldefluor assay, originally optimised to detect ALDH1 expression in HSCs [7] has been used to successfully enrich CSCs from breast cancer [8], leukemia [9], prostate cancer [10], colon cancer [11], bladder cancer [12] and liver cancer [13]. Because the Aldefluor substrate probably is not specific for this isoform [14], we refer only to ALDH-activity. ALDH-activity has also been associated with increased tumourigenicity in osteosarcoma [15]. Furthermore, several groups have reported that expression of ALDH is associated with high grade and poor prognosis in lung cancer [16], leukemia [9], ovarian cancer [17], breast cancer [8,18], colon cancer [11], prostate cancer [10], bladder cancer [12] and head and neck cancer [19]. ALDH expression has also been correlated with resistance to chemotherapy [19,20].

The surface molecule CD133, also known as AC133 and prominin-1, is expressed on normal stem cells [21] and on CSCs identified in a range of cancers [22], including cancer of the brain [23,24], colon [25,26], pancreas [27] and liver [28]. The majority of research concerning CD133 has been focused on epithelial cancers, but CD133 expressing-cells have also been observed in mesenchymal tumours. Recently, Tirino *et al.*, reported that CD133 is expressed in all of 21 primary bone sarcoma samples analysed (0, 21-7, 85%). Interestingly, the CD133^+ ^cells displayed CSC characteristics, such as increased ability to generate tumours *in vivo *and form spheres *in vitro*. The CD133^+ ^cells were also able to repopulate the culture with CD133^- ^cells, and were able to undergo differentiation [29]. Others have also reported that a subset of Ewing sarcoma primary tumours [30,31] and synovial sarcoma primary tumours [32] harbour CD133-expressing cells. In addition, several osteosarcoma cell lines contain subpopulations of cells (typically 3-5%), which are positive for CD133 [33].

Since the markers which are commonly used to isolate CSC populations do not uniquely identify CSCs, CSC enrichment can be improved by combining several markers. For instance, the enrichment of CSC populations from liver cancer cell lines using only CD133 was doubled when CD133 was used in combination with ALDH [13,28]. Similarly, Ginestier *et al *demonstrated that breast CSCs could be better enriched by combining Aldefluor with the markers CD44^+ ^CD24^- ^lin^-^, originally used by Al-Hajj and co-workers [34].

In this article we confirm that ALDH is expressed in liposarcoma primary material. Using a liposarcoma xenograft model system we show that ALDH is also expressed in this system, and that the combined use of Aldefluor and CD133 enables enrichment of a small cell population by flow cytometry. The Aldefluor^high ^CD133^high ^cells have CSC characteristics, such as increased ability to form spheroids in soft agar, and increased tumourigenicity *in vivo*.

## Materials and methods

### Ethics statement

The use of surplus patient material for cancer research is based on general written information and consent from the patients, combined with approval from the Regional Ethics Committee of Southern Norway for each project (Permit S-06133). All procedures involving animals were performed according to protocols approved by the National Animal Research Authority in compliance with the European Convention for the Protection of Vertebrates Used for Scientific Purposes (approval ID 1499, http://www.fdu.no).

### Immunohistochemical analyses of liposarcoma patient samples

Ten formalin-fixed and paraffin-embedded liposarcoma patient samples were obtained from the Department of Pathology at Oslo University Hospital (The Norwegian Radium Hospital). More specifically, the samples included 3 well-differentiated liposarcomas (grade 1-2), 3 de-differentiated liposarcomas (grade 4), 2 myxoid and round cell liposarcomas (grade 3-4) and 2 pleomorphic liposarcomas (grade 4). Four μm thick sections were made and processed for immunohistochemistry using the Dako EnVision™ Flex+ System (K8012, Dako Corporation) and Dakoautostainer. Sections were deparaffinized and epitopes unmasked using PT-Link (Dako) and EnVision™ Flex target retrieval solution, low pH. After blocking endogenous peroxidase with 0.03% hydrogen peroxide (H_2_O_2_) for 5 minutes, the sections were incubated with monoclonal mouse antibodies ALDH (1:3000, BD Transduction Laboratories™) and CD133/1 (AC133) (1:25, Miltenyi Biotec Inc.) over night at 4°C. Subsequently, the slides were incubated with EnVision™ Flex+ Mouse linker (15 min) and EnVision™ Flex/HRP enzyme (30 min). Tissue was stained for 10 minutes with 3'3-diaminobenzidine tetrahydrochloride (DAB) and then counterstained with haematoxylin, dehydrated and mounted in Diatex. Normal liver and the CaCO2 cell line (American Type Culture Collection No. HTB37 (Rockville, MD)) have been included as positive controls for ALDH and CD133, respectively. Negative controls included replacement of monoclonal antibodies with mouse myeloma protein of the same subclass and concentration as monoclonal antibodies. The immunoreactivity was evaluated according to the number of positively stained tumour cells (0 = none; 1 < 10%; 2 = 10 - 50%; 3 > 50%).

### Xenograft cell culture

The ATCC liposarcoma cell line SW872 (HTB92) (originally generated from a surgical specimen with histopathology of undifferentiated malignant liposarcoma.) was utilized to establish a xenograft in locally bred athymic NCR nu/nu mice (nude mice). The xenograft was then passaged to a new mouse before the tumour reached maximum 2 cm^3^. In order to extract cells from the xenografts, typically 6 - 8 tumors were minced in Hank's buffered saline solution (Invitrogen). The tissue-pieces were then incubated in 5 U/ml collagenase 4 (Worthington's) in DMEM:F12 (Gibco) for 45 minutes to 1 hour at 37°C. Cells were collected by passing the mixture through a 70 μm filter. The cells were subsequently maintained in either standard RPMI (Lonza) containing 10% fetal bovine serum (PAA laboratories Gmbh), 1× glutamax (Gibco) and 1 μg/ml penicillin/streptomycin (Lonza) or in stem cell (SC)-medium (70% mouse embryonic fibroblast conditioned medium (R&D systems) mixed with 30% of human embryonic stem cell medium (containing 20% "knock-out" serum replacement (Invitrogen), 1% non essential amino acids (Gibco), 4 ng/ml bFGF (Invitrogen), 0, 1 mM β-mercaptoethanol (Sigma), 1× glutamax (Gibco) in DMEM:F12 (Gibco))). The cells were maintained in culture for 10-14 days before analyses were performed. Adherent cells were dissociated when sub-confluent using TrypLE (Invitrogen).

### Phenotypic analysis and cell sorting using flow cytometry

Spheroid-shaped aggregates were dissociated by 45 minutes incubation in TrypLE (Invitrogen) at 37°C. Adherent cells were detached by a shorter incubation in TrypLE. Aldefluor staining (Stem Cell Technology) was performed at the concentration of 1 × 10^6 ^cells/ml Aldefluor assay buffer, according to the protocol recommended by the manufacturer. On all occasions the monoclonal mouse antibody TRA-1-85-APC (1:20, R&D systems), which recognizes an epitope found on all human cells, was included. On some occasions the cells were subsequently labeled with one of the following monoclonal mouse antibodies CD44-PE (1:10), CD90-PE (1:20), CD73-PE (1:10) (All from BD Pharmingen), CD105-PE (1:20, eBioscience), CD133/2(293C)-PE (1:10, Miltenyie Biotec. Inc), STRO-1-PE (1:20, Santa Cruz Biotec) or fibroblast growth factor receptor (FGFR)1 (M19B2) (1:100, Abcam). Cells stained with FGFR1 antibody were subsequently labeled with Alexa Fluor 647 donkey anti-mouse IgG (H+L) (1 μg/million cells, Invitrogen-Molecular Probes). The cells were incubated on ice for 40 minutes. The cells were then washed and filtered through a 40 μm filter, and subsequently analyzed or sorted by flow cytometry. Analyses were performed using a FACS ARIA-2 (Becton Dickenson). Viable singlets which were TRA-1-85^+ ^were sorted into the following four fractions: Aldefluor^high ^CD133^high^, Aldefluor^high ^CD133^low^, Aldefluor^low ^CD133^low ^and Aldefluor^low ^CD133^high^. The flow cytometry sorted cells were subject to viability analysis by trypan blue staining, before subsequent experiments were performed.

### Spheroid assay in soft agar

One thousand cells from each flow cytometry sorted subpopulation were plated in 0, 3% soft agar (Difco) in SC-medium in 35 mm non-adhesive dishes. Two hundred and fifty μl SC-medium was added once a week. Uniform spheroids of minimum 50 μm were counted approximately four weeks post plating.

### Adipocytic differentiation and Oil red O staining

Cells were grown in standard RPMI (Lonza) containing 10% fetal bovine serum (PAA laboratories Gmbh), 1× glutamax (Gibco) and 1 μg/ml penicillin/streptomycin (Lonza), supplemented with an adipocytic differentiation cocktail (50 μM Indomethacin, 1 μM Dexamethason, 0, 5 mM isobutyl-methyl-xanthine (IBMX)). Following 21 days in culture, the cells were fixed in 70% ethanol and subsequently stained in 0, 3% oil red O, and analyzed in a fluorescence microscope (Olympus IX81). Lipid droplets in mature adipocytes appeared red.

### *In vivo *tumourigenicity

Serial dilutions (100 - 25 000 cells) of each sorted subpopulation were injected subcutaneously into the flanks of locally bred athymic NCR nu/nu mice (nude mice). TRA-1-85^+ ^(human specific epitope) cells were injected as unselected controls. The cells were diluted in a final volume of 100 μl DMEM:F12 (Gibco). Viability of the injected cells was confirmed by trypan blue (Sigma) staining prior to injection.

## Results

### Aldehyde dehydrogenase is expressed in primary human liposarcomas

**Figure 1:**
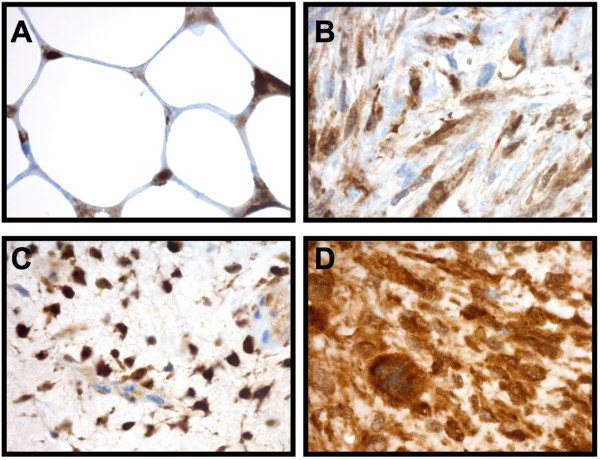
**ALDH1 expression in liposarcoma patient samples**. ALDH1 was expressed in 10 out of 10 primary liposarcoma tumors analysed by immunohistochemsitry. **(A) **Well differentiated-, **(B) **De-differentiated-, **(C) **Myx/roundcell- and **(D) **Pleomorphic-liposarcoma.

Immunohistochemical analyses of ALDH1 expression in liposarcoma patient samples confirmed that 10 out of 10 samples expressed ALDH1. More specifically, 8 out of 10 samples expressed ALDH1 in more than 50% of the tumour cells. One patient sample displayed ALDH1 expression in 10 - 50% of the tumour cells, and for one patient sample, less than 10% of the tumour cells were ALDH1 positive (Figure 1, Table 1). The samples represented a range of liposarcoma sub-types (well-differentiated, de-differentiated, myxoid/round celled and pleomorphic liposarcoma). We were not able to find any correlations between particular liposarcoma subtypes and the level of ALDH1 expression in this small and diverse panel.

**Table 1:** CD133 and ALDH1 expression in liposarcoma patient samples.

Diagnosis	Tumour site	Age	Pre-treatment	Grade	CD133	ALDH1	
						**Cytoplasm**	**Nucleus**

Well-diff.	Retroperitoneal	36	No treatment	1	0	3	3

Well-diff.	Retroperitoneal	57	No treatment	2	0	3	3

Myx/roundcell	Thigh	41	No treatment	3	0	3	3

Myx/roundcell	Thigh	79	Chemotherapy	4	0	3	3

De-diff.	Thigh	79	No treatment	4	0	3	3

De-diff.	Retroperitoneal	60	No treatment	4	0	1	1

Well-diff. Comp *	Retroperitoneal	64	No treatment		0	3	3

De-diff. Comp*	Retroperitoneal	64	No treatment	4	0	3	3

Pleomorphic	Truncus	67	No treatment	4	0	3	3

Pleomorphic	Retroperitoneal	58	No treatment	4	0	3	2

Well-diff.	Leg	31	No treatment	1	0	2	2

Ten liposarcomas diagnosed as well-differentiated (well-diff.), myxoid/roundcelled (myx/roundcell), de-differenitated (de-diff) or pleomorphic were included in the analyses. Tumour location, patient age, treatment prior to sample collection and tumour grade are also displayed. CD133 and ALDH1 expression was scored as follows: 0 = negative, 1 = less than 10% of the tumour cells scored positive, 2 = 10-50% of the rumour cells scored positive, 3 = more than 50% of the tumour cells scored positive. *For one of the tumors, a de-differentiated and a well-differentiated component was analysed.

### Aldehyde dehydrogenase is expressed in the liposarcoma xenograft SW872

**Figure 2:**
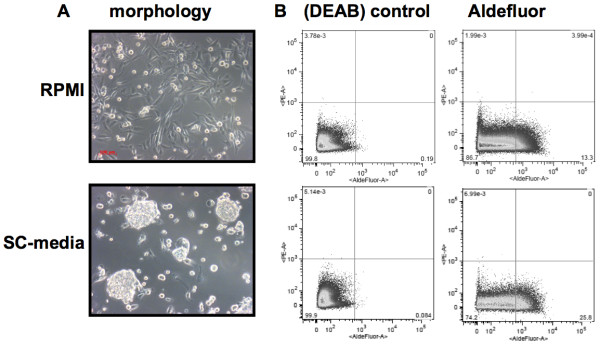
**Characterisation of SW872 xenograft-derived cells following culturing in RPMI or stem cell medium**. **(A) **Different morpholgy was observed dependent on the culturing medium. The cells appeared adherent when cultured in standard RPMI supplemented with fetal bovine serum (upper panel) and grew as detached spheroids when cultured in SC-medium (lower panel). **(B) **Flow diagrams are shown for control (DEAB) samples (left), and Aldefluor sample (right). 26% of the cells displayed Aldefluor activity when maintained in SC-medium (lower panel), compared to 13% of the cells when maintained in RPMI (upper panel). Aldefluor intensity is displayed along the X-axis. **(C) **Average Aldefluor^high ^cells following culturing in SC-medium (35%) (black) (n = 10) or RPMI (11%) (grey) (n = 3).

Having confirmed that ALDH1 is indeed expressed in human liposarcomas, we wanted to investigate whether liposarcoma ALDH-positive cells could be associated with CSC activity. We preferred to use a xenograft model, because the passing of the xenograft from mouse to mouse ensures that the growth conditions are physiological and that tumour initiating cells are present. Aldefluor analysis of cells extracted from the SW872 liposarcoma xenograft showed that the SW872 xenograft cells, like the liposarcoma patient samples, displayed ALDH activity (11% of the cells were Aldefluor^high^: Figure 2B), making xenograft-derived SW872 cells a suitable model for further analyses of ALDH-positive cells.

### Cellular growth pattern, morphology and expression of stem cell markers are affected by the culturing medium

In order to maintain the extracted cells in a culturing medium best suited for enriching CSCs, we first investigated the effect of different culturing media on the expression of ALDH and other stem cell markers. The extracted xenograft cells were therefore maintained for 10-14 days in either standard RPMI medium containing fetal bovine serum (RPMI) or stem cell medium (SC-medium) containing "knock-out" serum replacement, mouse embryonic fibroblast (MEF) conditioned medium and basic fibroblast growth factor (bFGF), commonly included in embryonic stem cell medium to prevent differentiation [35]. The cellular morphology was highly dependent on the culturing medium. Cells maintained in RPMI exhibited an adherent morphology and cells maintained in SC-medium attached to each other and grew as large aggregated spheroids in 3D suspension (Figure 2A). Cellular growth as spheroids in suspension has previously been associated with stem-ness and tumor initiating activity [36,37]. Interestingly, when the cells had been maintained in SC-medium, a larger percentage of the cells displayed ALDH activity (average 35% Aldefluor positive cells), compared to the average 11% observed when the cells were maintained in RPMI. The ALDH inhibitor diethylamino-benzaldehyde (DEAB) could block this activity (Figure 2B). Furthermore, when cells were initially incubated in RPMI for 6 days and then transferred into SC-medium for the remaining period, the percentage of cells displaying Aldefluor activity increased (data not shown). These findings indicate that the cells comprise a degree of plasticity, and that cells which have the capacity to become more stem-like may do so in the presence of growth factors in the SC-medium. For instance, FGF signaling is implicated in regulation of self-renewal and differentiation. Since bFGF binds to and activates FGF Receptor 1 (FGFR1) [38], we decided to investigate FGFR1 membrane expression in SW872. Interestingly, we found that FGFR1 was highly expressed in the SW872 cell line. Furthermore, expression of FGFR1 was induced during culturing of xenograft-derived SW872 cells in SC-medium (86, 8%) compared to culturing in RPMI (62, 8%) (Table 2), indicating that activation of FGFR1 may result in expansion of CSCs. According to the CSC theory, the CSC population represent a small sub-population within the tumor [2]. In keeping with this theory, others have shown that a smaller, better enriched CSC population is isolated by flow cytometric cell sorting when combining the Aldefluor assay with antibody staining of CSC surface antigens [8,13]. Thus, we would expect the large Aldefluor^high ^cell population observed after culturing the cells in SC-medium to be heterogeneous, and the CSCs to represent a smaller population within the Aldefluor^high ^cell population.

**Table 2:** Phenotypic analyses of SW872.

SW872	Xenograft-derived cells	Xenograft-derived cells	Cell line
**Surface marker**	**SC-medium**	**RPMI**	**RPMI**

FGFR-1^high^	86, 8	62, 8	43, 4

Aldefluour^high^	35, 0	11, 0	0, 2

CD90^high^	93, 3	89, 4	41, 6

CD44^high^	97, 9	98, 3	99, 9

CD105^high^	97, 5	95, 6	82, 1

STRO-1^high^	0, 5	0, 7	0, 3

CD73^high^	2, 6	4, 4	27, 4

CD133^high^	0, 6	0, 3	0, 3

Aldefluour^high ^CD90^high^	41, 9	5, 5	ND

Aldefluour^high ^CD44^high^	39, 8	3, 7	ND

Aldefluour^high ^CD105^high^	44, 8	2, 8	ND

Aldefluour^high ^STRO-1^high^	0, 2	0, 1	ND

Aldefluour^high ^CD73^high^	1, 3	3, 2	ND

Aldefluour^high ^CD133^high^	0, 1	0	ND

The table displays the average percentages of cells scored as Aldelfuor^high^, surface marker^high ^or Aldelfuor^high ^surface marker^high ^in the respective culturing medium, as determined by flow cytometry (minimum two parallels were performed). The SW872 cell line was not subjected to co-staining as only 0, 2% of the cells were scored as Aldefluor^high^.

In the case of liposarcoma, a likely cell of origin for the CSC would be a mesenchymal progenitor or stem cell (MSC). To our knowledge, no surface marker is known to uniquely identify MSCs, so we first tested the cell surface expression of the following markers, which are known to be expressed on MSCs: CD44, CD73, CD105, CD90 and STRO-1 [39,40]. We also included the stem cell and CSC marker CD133 in our screen [41]. In addition we performed phenotypic analyses of the original SW872 cell line (Table 2). With the aim to identify a small Aldefluor^high ^surface marker^high ^(double-positive) cell population, we performed the Aldefluor assay in combination with antibody staining against each surface marker. When testing Aldefluor in combination with CD90, CD44 or CD105 staining, we found that dual expression was observed in a small percentage of the cells following culturing in RPMI. The percentage of double-positive cells increased dramatically to approximately 40% due to an increasing number of cells expressing ALDH when the cells were maintained in SC-medium (Table 2). Next we tested Aldefluor in combination with STRO-1 or CD73 staining, and found that a relatively small percentage of cells were double-positive, independent of medium. Finally, we tested Aldefluor in combination with CD133 and found that no cells were double-positive when the cells were incubated in RPMI. However, interestingly we found that 0, 1% of the cells displayed an Aldefluor^high ^CD133^high ^phenotype when maintained in SC-medium. Because CSCs are expected to represent a small fraction of the tumour cells, using CD90, CD44 or CD105 in combination with Aldefluor would not be likely to result in sufficient enrichment of CSCs. On the contrary, CD73, STRO-1 and CD133 might be suitable as CSC-markers, since these markers, when combined with Aldefluor, identified a small population of SW872 xenograft-derived cells. The Aldefluor^high ^CD133^high ^phenotype was consistently observed in a small population (0, 1 - 1, 7%, n = 9) of cells cultured in SC-medium. The Aldefluor^high ^CD133^high ^subpopulation disappeared when cells were cultured in RPMI, indicating that the combined expression of these two stem cell markers had been induced by factors in the stem cell media. Subsequently, we were interested in evaluating whether cells with an Aldefluor^high ^CD133^high ^phenotype comprised a CSC-potential. We therefore decided to perform further characterization of this subpopulation with respect to CSC abilities.

### Aldefluor^high ^CD133^high ^cells have an enhanced ability to form spheroids

**Figure 3:**
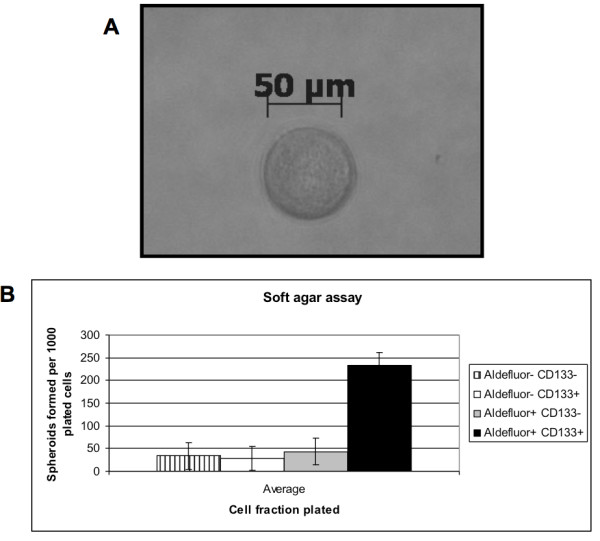
**Aldefluour^high ^CD133^high ^SW872 xenograft-derived cells form spheroids more effciently in soft agar**. **(A**) Typical round-shaped spheroid of 50 μm formed from single Aldefluour^high ^CD133^high ^cell. **(B) **Aldefluor^high ^CD133^high ^cells formed spheroids with a frequency of up to 1 out of 4 cells (n = 4).

Using flow cytometry, we isolated 4 subpopulations based on ALDH and CD133 expression. In order to investigate the different cell population's stem-like ability to self-renew, we performed spheroid assays in soft agar. The Aldefluor^high ^CD133^high ^cell population generated well-defined, round spheroids of approximately 50 μm in size (Figure 3A), at a frequency of up to 1 out of 4 cells. All the other three subpopulations generated spheroids at a significantly lower frequency (Figure 3B).

### Aldefluor^high ^CD133^high ^cells have the ability to differentiate into adipocytes

**Figure 4:**
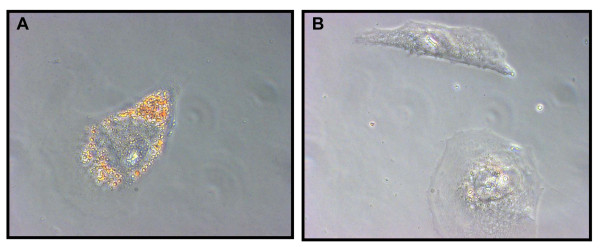
**Aldefluour^high ^CD133^high ^SW872 xenograft-derived cells differentiate into adipocytes**. **(A**) Accumulation of lipid droplets indicative of mature adipocytes was observed following culturing of Aldefluour^high ^CD133^high ^sorted SW872 cells in medium supplemented with adipocytic differentiation cocktail (visualized by oil red O staining). **(B) **Aldefluour^high ^CD133^high ^sorted SW872 cells did not differentiate as efficiently when maintained in standard RPMI medium.

According to the theory, a CSC has the ability to both generate more CSCs through self-renewal, and to undergo partial differentiation generating heterogeneous cancer cells, which make up the bulk of the tumour. Liposarcomas are in part composed of adipocytes and a potential liposarcoma CSC should therefore have the capacity to differentiate into adipocytes. When culturing the sorted cell populations in the presence of an adipocytic differentiation cocktail, we found that the Aldefluor^high ^CD133^high ^cells were able to differentiate into mature adipocytes more efficiently than the other sorted cell populations (Figure 4).

### Aldefluor^high ^CD133^high ^cells form tumors more efficiently *in vivo*

One of the hallmarks of CSCs is the increased ability to form tumors *in vivo*. Following flow cytometry, serial dilutions (100, 1000, 5000 and 25 000 cells) of the four sorted subpopulations were injected into immunodeficient nude mice. The Aldefluor^high ^CD133^high ^cells produced tumors more efficiently in nude mice compared to the other sorted cell populations (Table 3). As few as 100 Aldefluor^high ^CD133^high ^cells were sufficient to generate tumors in 14% of the mice, whilst no tumors were formed when the other subpopulations were injected at this cell dilution. When injecting 5000 cells of the Aldefluor^high ^CD133^high ^subpopulation, the majority of the injections (66%) resulted in tumour formation. We were unable to obtain sufficient number of cells to inject 25 000 Aldefluor^high ^CD133^high ^cells.

**Table 3:** *In vivo *tumourigenicity of SW872 xenograft-derived subpopulations.

Cells injected	25 000	5 000	1000	100
Aldefluor^high ^CD133^high^	ND	2/3	4/14	2/14
Aldefluor^high ^CD133^low^	2/12	3/16	2/14	0/14
Aldefluor^low ^CD133^high^	0/6	1/16	0/18	0/16
Aldefluor^low ^CD133^low^	2/14	7/14	7/18	0/14
TRA-1-85^+ ^(Control)	2/12	2/12	8/16	0/10

The table displays the total number of tumors formed, divided by the total number of injections performed. 100 - 25 000 cells of each group were injected subcutaneously into immunodeficient mice. Tumourigenicity was not determined (ND) for 25 000 Aldefluor^high ^CD133^high ^cells. TRA-1-85^+ ^represent viable, single SW872 cells. The results are accumulated over three individual experiments.

## Discussion

In this study, we initially chose to focus on Aldefluor as a CSC marker for several reasons. Firstly, the Aldefluor assay has been used to successfully isolate CSCs from several malignancies [8-13,15]. Secondly, we found ALDH1 a clinically relevant marker, identifying subpopulations of cancer cells in all liposarcoma patient samples analyzed. ALDH expression has proven a useful marker for cancers of several tissues [8-12,16-19,42]. Thirdly, the Aldefluor assay is less cytotoxic compared to other CSC isolation methods (e.g. side population assay), and since an intact cell membrane is required, only viable cells are isolated. Although the analyses of these phenotypes require separation of individual cells and short term *in vitro *culturing, we chose to use a xenograft-derived cell model to better mimic the 3D growth conditions and stroma interactions of *in vivo *human tumors. Furthermore, the continuous passaging of the xenograft ensured the presence of tumour-initiating cells. Moreover, *in vitro *conditions are not necessarily favorable for maintaining stem-ness, and we therefore compared the effects of two different culturing medium. Morphological observations and Aldefluor analyses of the SW872 xenograft-derived cells maintained in SC or RPMI medium indicated that the SC-medium was the more favourable for maintaining/inducing the CSC phenotype *in vitro*. The cells displayed an adherent cellular morphology when maintained in RPMI, while the cells grew as detached, round "spheroid"-aggregates when the cells were maintained in SC-medium, a growth-pattern that has been associated with stem-ness [23,43]. Furthermore, the fact that the percentage of cells which displayed ALDH activity was significantly higher when the cells were maintained in SC-medium also indicated that the SC-medium is favorable for enrichment of CSCs. Moreover, the observed increase in number of cells displaying high Aldefluor activity following a change of medium from RPMI to SC, indicates that a subpopulation of the bulk cells have a potential to become more "stem-like" in response to certain stimuli. It is likely that the 3D cell-cell contacts, as well as the mixture of growth factors in the SC-medium maintain and induce CSC self-renewal. Since a large percentage of the SW872 cells express FGFR1, and the percentage of cells expressing FGFR1 is further increased following culturing in SC-medium (containing bFGF), it is possible that CSCs are enriched through FGFR activation.

A large percentage of the SW872 liposarcoma xenograft-derived cells were Aldefluor positive, making it unlikely that ALDH as a single marker could be used to identify a pure CSC population. Others have shown that the use of Aldefluor in combination with other stem cell markers improves the enrichment of CSCs [8,13,42]. A likely cell of origin for the sarcoma-CSC is an MSC-like stem or progenitor cell. However, since no markers are known to uniquely identify MSCs, we investigated a range of markers expressed on MSCs. We also included the stem cell and CSC marker CD133 [22-28,31]. Although several of the Aldefluor^high ^surface marker^high ^subpopulations identified in this screen might enrich for CSCs, the Aldefluor^high ^CD133^high ^cells seemed particularly promising. This small subpopulation was only observed in the 3D spheroid culture (SC-medium), indicating that the phenotype was either selectively induced by factors in the SC-medium, or was dependent on the growth pattern.

**Figure 5:**
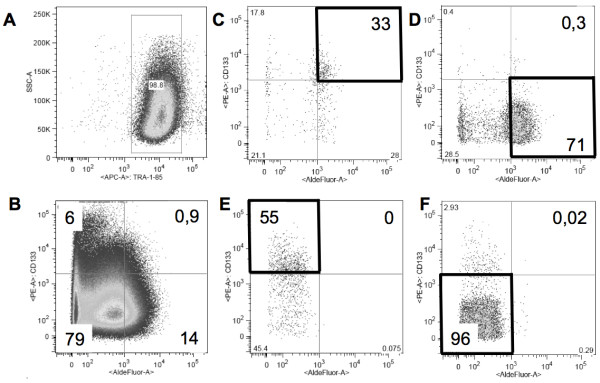
**Flow cytometry and purity testing of sorted fractions**. **(A) **Viable, single, human (TRA-1-85+) SW872 xenograft-derived cells (98, 8%) were sorted on the basis of **(B) **Aldefluor (X-axis) and CD133 (Y-axis) activity. In this representative experiment the subpopulations in the culture were as follows: 79% Aldefluor^low ^CD133^low^, 6% Aldefluor^low ^CD133^high^, 14% Aldefluor^high ^CD133^low ^and 0, 9% Aldefluor^high ^CD133^high^. The 4 flow sorted subpopulations were subject to subsequent purity testing: **(C) **Aldefluor^high ^CD133^high^: 33% pure, **(D) **Aldefluor^high ^CD133^low^: 71% pure and containing 0, 3% potential CSCs **(E) **Aldefluor^low ^CD133^high^: 55% pure and **(F) **Aldefluor^low ^CD133^low^: 96% pure.

The functional analysis of the sorted subpopulations of SW872 cells demonstrated that the Aldefluor^high ^CD133^high ^cells had a highly increased ability to form spheroids in soft agar, indicating that these cells have an increased ability to self-renew compared to the other sorted cell populations. Interestingly, the Aldefluor^high ^CD133^high ^cells had higher capacity to differentiate into adipocytes. Whether the Aldefluor^high ^CD133^high ^cells have multi-lineage potential was not tested. However, since the Aldefluor^high ^CD133^high ^CSC is likely to originate from a MSC, it would be interesting to investigate the ability of these cells to differentiate into other mesenchymal cell types, such as osteoblasts and chondrocytes. Our *in vivo *tumourigenicity assay showed that the Aldefluor^high ^CD133^high ^subpopulation overall generated tumors more efficiently compared to the other subpopulations when injected subcutaneously into nude mice, in particular at low cell numbers. However, at higher cell numbers tumors were also generated by some of the other subpopulations. Re-analyses of each isolated subpopulation was done by a second round of flow cytometry to determine the purity of the isolated fractions. As demonstrated in Figure 5, the Aldefluor^high ^CD133^high ^subpopulation was only enriched to 33% purity, with a large percentage of tumour cells from the other subpopulations "diluting" the CSC population. The Aldefluor^high^CD133^low ^flow sorted subpopulations was clearly "contaminated" with a few Aldefluor^high ^CD133^high ^cells, which likely contributed to tumour formation at high cell numbers. The purity of the flow sorting may be compromised by variability in expression and staining, but also by inherent "noise" in the flow sorter. The fact that the Aldefluor^high ^CD133^high ^cell population is only enriched also partly explains why tumors are not formed in all Aldefluor^high ^CD133^high ^injections. Furthermore, when separating the cells into subpopulations, the CSCs may lack the support of cells that are required to make up a "niche" *in vivo*.

ALDH1 was expressed in all the liposarcoma patient samples analyzed by IHC. Although the level of expression varied from less than 10% of the tumor cells expressing ALDH1 to more than 50% of the tumor cells expressing ALDH1, we were not able to correlate the differences in level of expression with any particular factors; neither sub-type, tumor location, patient age or tumor grade. Furthermore, we were unable to confirm CD133 expression in the same panel (data not shown). There are several problems associated with CD133 immunohistochemical expression analysis [41]. Several groups have reported that the antibodies binding CD133 detect only the glycosylated epitopes [44]. However, Kemper *et al *demonstrated that bacterially expressed CD133 or CD133 glycosylation mutants were indeed recognized by the CD133 antibody AC133 used here. Instead the authors concluded that the accessibility of the AC133 epitope varied [45]. Although we cannot confirm CD133 expression in our primary material, CD133 might still be present on the surface, but undetectable by the AC133 antibody due to epitope masking. Alternatively, expression of CD133 may only be present in very few cells or at a frequency below the detection level of immunohistochemistry. This is consistent with Suva *et al *and Tirino *et al *who both show that CD133 positive cells are extremely rare in sarcoma patient material [29,31].

## Conclusion

In conclusion, we have demonstrated that ALDH1 is expressed in liposarcoma patient samples, although we were unable to confirm CD133 expression in the same material. We have performed extensive phenotypic analyses of liposarcoma xenograft-derived cells using Aldefluor and surface markers, and as a result identified a CSC-like subpopulation of cells expressing both ALDH and CD133 when cultured as spheroids in SC-medium. Furthermore, we have demonstrated that this phenotype is associated with stem-like abilities, such as increased ability to self-renew and to form tumours in immunodeficient mice. Although it remains to be validated whether Aldefluor and CD133 in combination can be used to isolate CSCs from liposarcomas and sarcomas in general, these markers have proven useful for isolating CSCs across tumor types [13], and may be used as targets for novel CSC-specific therapies. Ongoing work includes specifically targeting and killing the CSC population in our model system.

## List of abbreviations

CSC: cancer stem cell; bFGF: basic fibroblast growth factor; FGFR: fibroblast growth factor receptor; HSC: hematopoietic stem cell; MSC: mesenchymal stem cell; ALDH: aldehyde dehydrogenase.

## Competing interests

The authors declare that they have no competing interests.

## Authors' contributions

EWS, EM and OM designed the study and wrote the manuscript. EWS, ABW and SL performed the practical work, apart from the flow cytometry which was done by RC and the immunohistochemistry performed by RH. RH and BB performed pathological analyses. All authors read and approved the final manuscript.
